# Differential Serum Cytokine Profiles in Aborting and Subclinically Infected Pregnant Mares with Equine Herpesvirus Type 1

**DOI:** 10.3390/ani16162455

**Published:** 2026-08-07

**Authors:** Ömer Deniz, Gencay Ekinci, Mehmet Çitil, Ali Cesur Onmaz, Fatih Mehmet Derelli, Mustafa Cem Timur, Köksal Altınbay, Alper Mete, Ersin Yeğen, René van den Hoven, Francesco Fazio, Francesca Aragona

**Affiliations:** 1 Department of Internal Medicine, Faculty of Veterinary Medicine, Kastamonu University, Kastamonu 37150, Türkiye; odeniz@kastamonu.edu.tr; 2 Department of Internal Medicine, Faculty of Veterinary Medicine, Erciyes University, Kayseri 38280, Türkiye; gencayekinci@gmail.com (G.E.); mcitil@hotmail.com (M.Ç.); aonmaz@gmail.com (A.C.O.); 3 Jockey Club of Türkiye, Izmit Central Covering Station, İzmit 41310, Türkiye; fderelli@gmail.com; 4 Jockey Club of Türkiye, Equine Health and Veterinary Services, Head Office, Bakırköy, Istanbul 34144, Türkiye; mctimur@tjk.org (M.C.T.); koksalaltinbay@gmail.com (K.A.); 5 Jockey Club of Türkiye, Istanbul Equine Hospital, Istanbul 34144, Türkiye; alpermete1985@yahoo.com; 6 Department of Virology, Veterinary Faculty, Istanbul University-Cerrahpasa, Buyukcekmece, Hadimkoy, Istanbul 34555, Türkiye; ygnersin@gmail.com; 7 Department of Companion Animals and Horses, Equine University Clinic for Internal Medicine and Infectious Diseases, Veterinary University of Vienna, Veterinärplatz 1, A-1210 Vienna, Austria; prof.renevandenhoven@gmail.com; 8 Department of Veterinary Science, University of Messina, Via Giovanni Palatucci, 98168 Messina, Italy; fraragona@unime.it

**Keywords:** abortion, horse, cytokines, pregnant mares, equine herpesvirus type 1, immunology

## Abstract

Equine herpesvirus type 1 (EHV-1) is a major cause of reproductive loss in mares. This cross-sectional field study evaluated serum concentrations of interleukin-2 (IL-2), interleukin-10 (IL-10), interferon-alpha (IFN-α), and interferon-gamma (IFN-γ), together with the IL-10/IL-2 and IFN-γ/IL-10 ratios, in 26 vaccinated pregnant Thoroughbred mares. The analytical cohort comprised healthy, EHV-1 PCR-negative mares (HM, n = 10), EHV-1 PCR-positive mares sampled within 4 h after fetal expulsion at approximately the sixth month of gestation (AM, n = 9), and EHV-1 PCR-positive pregnant mares without clinically relevant signs (WCS, n = 7). EHV-1 DNA was detected using an EHV-1-specific real-time PCR assay, and serum cytokine concentrations were measured using equine-specific ELISA kits. Serum IFN-α, IFN-γ, and IL-2 concentrations were significantly higher in both AM and WCS mares than in HM mares (all Holm-adjusted *p* < 0.001), whereas no statistically significant differences in these cytokines were observed between the AM and WCS groups. IL-10 concentrations were significantly higher in WCS mares than in both HM and AM mares (both adjusted *p* < 0.01), and HM and AM mares did not differ significantly. The IL-10/IL-2 ratio was highest in HM mares, intermediate in WCS mares, and lowest in AM mares, with significant differences among all pairwise comparisons (adjusted *p* ≤ 0.019). Conversely, the IFN-γ/IL-10 ratio was highest in AM mares, intermediate in WCS mares, and lowest in HM mares, again with significant differences among all pairwise comparisons (adjusted *p* ≤ 0.044). These findings indicate substantial antiviral immune activation in both EHV-1-positive groups, with a comparatively stronger regulatory immune component in WCS mares and a predominantly pro-inflammatory profile in mares sampled after abortion.

## Simple Summary

Equine herpesvirus type 1 (EHV-1) is a major cause of abortion in pregnant mares, with reproductive losses thought to be largely driven by the host immune response. This study evaluated serum concentrations of interleukin-2 (IL-2), interleukin-10 (IL-10), interferon-alpha (IFN-α), and interferon-gamma (IFN-γ) in three groups of mares: healthy pregnant mares (HM), EHV-1 PCR-positive mares that aborted at the sixth month of gestation (AM), and PCR-positive pregnant mares without clinical signs (WCS). EHV-1 DNA was detected by real-time PCR in blood and nasopharyngeal swabs and, in mares that aborted, in fetal and placental tissues. Cytokine concentrations were measured using equine-specific ELISA kits. Both EHV-1-positive groups showed marked antiviral cytokine activation. Samples from the PCR-positive mares without clinical signs (WCS) had elevated IFN-α, IFN-γ, IL-2, and IL-10 concentrations, suggesting that IL-10 may contribute to immune regulation and the absence of clinical signs. Cytokine ratio analysis further demonstrated lower IL-10/IL-2 and higher IFN-γ/IL-10 ratios in aborted mares compared with healthy controls, reflecting an imbalance toward inflammation. These findings indicate that EHV-1 infection induces distinct immunological profiles and suggest that serum cytokine ratios may provide additional information for characterizing immune responses in EHV-1-infected pregnant mares.

## 1. Introduction

Equine herpesvirus type 1 (EHV-1) is a highly prevalent alphaherpesvirus of major clinical and economic importance in horses worldwide, and serves as a major cause of abortion, neonatal mortality, and neurological disease, including equine herpesvirus myeloencephalopathy (EHM) [1]. Infection during gestation can lead to placental and fetal involvement, resulting in abortion outbreaks and substantial reproductive losses in breeding populations. EHV-1 can establish latency and subsequently reactivate, thereby maintaining an ongoing risk within affected herds [2].

The host immune response to EHV-1 involves complex interactions between innate and adaptive components [1]. Early innate responses include the production of type I interferons (e.g., IFN-α) and chemokines in the upper respiratory tract, which can limit viral replication, reduce cell-associated viremia, and shape subsequent adaptive immune responses [3,4,5,6]. T cell-mediated responses, including IFN-γ production, play a key role in controlling viral infection [6,7,8,9,10,11]. Distinct immunophenotypes have been reported, with increased expression of regulatory cytokines such as interleukin-10 (IL-10) associated with less favorable clinical outcomes, including a higher risk of EHM and potentially reproductive failure [10,11,12]. The immunological coordination relies on a highly regulated cytokine network, within which interleukins serve as principal signaling molecules [13]. Equine gestation involves dynamic, stage-specific shifts between pro-inflammatory and anti-inflammatory immune environments at the maternal–fetal interface [14]. Elevated expression levels of pro-inflammatory cytokines such as IL-2, IL-1β and IFN-γ are observed in the endometrium to coordinate local tissue remodeling, enhance vascular permeability, and facilitate maternal–fetal cellular crosstalk [15,16]. IL-1α, IL-1β, and IL-6 actively modulate endometrial prostaglandin synthesis, which is tightly regulated by maternal progesterone to downregulate the transcription of key interleukin receptors, thereby preventing premature luteolysis and sustaining pregnancy [13]. As pregnancy progresses past the implantation window, the immunological profile shifts toward an anti-inflammatory state to protect the fetus. This phase may be associated with an increase in IL-4 and IL-10 levels, alongside the expansion of regulatory T cells that suppress maternal cytotoxic responses [14,16]. Despite these findings, systemic cytokine responses in pregnant mares remain poorly understood. Pregnancy induces immunologic adaptations that may affect host responses to viral infections. The interaction between pregnancy-associated immune modulation and EHV-1 pathogenesis is not fully understood, yet it is likely to influence biomarker profiles during infection. A vaccinated population should be regarded as representative of real-world conditions, in which vaccination is widely used as a standard preventive measure [17]. Vaccination is known to reduce the severity of clinical disease and the risk of virus transmission, although it does not consistently prevent infection, viral shedding, or EHV-1-associated abortion. Moreover, vaccine-induced immune responses may influence the magnitude and duration of viral replication, clinical presentation, and diagnostic test results, thereby complicating the interpretation of observed associations [17]. Few field studies have assessed systemic cytokine profiles in pregnant mares exposed to EHV-1 under natural conditions. Therefore, the aim of this study was to compare serum cytokine concentrations among groups of pregnant mares with different EHV-1 infection outcomes. We hypothesized that mares that aborted would have a more pronounced pro-inflammatory response than both healthy mares and PCR-positive mares without clinically relevant signs (WCS).

## 2. Materials and Methods

### 2.1. Study Design

The experimental protocol was approved by the Kastamonu University Animal Experiments Local Ethics Committee (Decision No. 2025/16). This study was conducted in pregnant Thoroughbred mares with a mean body weight of 550 ± 20 kg during the 2021 breeding season on a breeding farm, with the owner’s informed consent obtained via signed agreement. According to farm records, 1144 horses entered the breeding farm during the 2021 breeding season, and 49 abortions were recorded. These figures are provided solely as farm-level epidemiological context and did not constitute the denominator from which the analytical cohort was sequentially selected. The study was not designed to estimate the farm-level prevalence of abortion or EHV-1 infection. The analytical cohort comprised a convenience sample of 26 multiparous pregnant Thoroughbred mares aged 6 to 15 years from the same insemination period and at comparable stages of gestation. No mares were excluded after inclusion in the analytical cohort, and complete clinical, PCR, and serum cytokine data were obtained for all 26 mares. Inclusion was based on the availability of complete clinical, PCR, and serum cytokine data, together with uniform documented vaccination exposure before sampling. A formal eligibility-screening cascade involving all 1144 horses was not performed. The mares were monitored by the same veterinarian according to their insemination dates and ultrasound scans to rule out potential confounding factors. The animals were housed in individual stalls with daily access to adjacent pasture, for varying durations depending on the season and weather conditions, as managed by experienced stable staff. The diet consisted of pasture, meadow hay, and commercial concentrates, with water provided ad libitum. All mares were routinely vaccinated against EHV-1 and equine herpesvirus-4 (EHV-4) using a commercially available inactivated vaccine (Duvaxyn EHV-1,4; Zoetis, Madrid, Spain). Vaccination was carried out according to the farm protocol at the fifth month of gestation. To ensure consistency in vaccination status, only mares that had received at least one dose of the EHV-1/4 vaccine during the relevant year were included; unvaccinated animals were excluded. All mares included in the study had received the same number of vaccine doses before sample collection. Specifically, all mares had received the scheduled inactivated EHV-1/EHV-4 vaccine during the fifth month of the current pregnancy, and none had received the scheduled seventh-month booster at the time of sampling. Therefore, vaccination exposure did not differ among the study groups and was controlled through the study inclusion criteria rather than through statistical adjustment. Among the 26 mares included in the analytical cohort, 9 aborted at 180 ± 7 days of gestation and were assigned to the AM group. All mares in the AM group were sampled within 4 h after fetal expulsion, and the post-abortion serum samples were used for cytokine analysis. Of the remaining mares, 7 were EHV-1 PCR-positive without clinically relevant signs and were assigned to the WCS group, whereas 10 were EHV-1 PCR-negative and clinically healthy and were assigned to the HM group. Mares in the WCS and HM groups were sampled during the same outbreak investigation period (Table 1). Because the mares originated from the same insemination period, the HM and WCS mares were at gestational stages comparable to those of the AM mares, corresponding approximately to the sixth month of gestation. Sampling was triggered by the abortion outbreak and was not performed at a scheduled vaccination time point; it occurred at least approximately 3 weeks or more after the fifth-month vaccination and before the scheduled seventh-month booster. Therefore, cytokine measurements in the AM group represent the post-abortion immune state and may reflect both the immune response to EHV-1 infection and the acute inflammatory response associated with abortion and placental tissue disruption. These measurements should not be interpreted as pre-abortion baseline or prognostic values.

### 2.2. Sample Collection

Blood samples were collected from all mares (n = 26) into EDTA tubes for whole-blood analysis and into 5 mL clot-activator tubes for serum preparation. Blood samples from the WCS and HM groups were collected in the early morning hours considering the circadian oscillation of immune and inflammatory cytokines which defines a potential susceptibility window for viral exposure [9,18]. EDTA blood samples were used for EHV-1 DNA detection by real-time PCR. Simultaneously, nasopharyngeal swabs were taken from all mares. Additionally, tissue samples from the liver, thymus, spleen, and lung of aborted fetuses, together with placental tissue samples, were collected for virological analysis.

### 2.3. EHV-1 PCR Analysis

For virological analyses, EDTA blood and nasopharyngeal swabs collected from all mares were processed immediately upon arrival at the laboratory. Pooled fetal organ tissue samples comprising liver, thymus, spleen, and lung, together with placental tissue samples, were processed only from mares in the AM group. Nucleic acid extraction was performed using an automated magnetic bead-based platform (TanBead Maelstrom 4810, Taiwan Advanced Nanotech Inc., Taoyuan, Taiwan) and a dedicated viral extraction kit (TanBead OptiPure Viral Auto Tube, Taiwan Advanced Nanotech Inc., Taoyuan, Taiwan), in accordance with the manufacturer’s instructions. EHV-1 DNA detection was performed using an EHV-1-specific real-time PCR assay incorporating WOAH-recommended primer and probe sequences [19]. Primer and probe sequences are provided in Table 2. PCR reactions were prepared in a total volume of 20 µL, consisting of 10 µL of 2× qPCR master mix, 0.8 µL of each forward and reverse primer (final concentration 400 nM), 0.4 µL of probe (200 nM), 5 µL of extracted nucleic acid, and 3 µL of nuclease-free water [20]. Amplification conditions included an initial denaturation step at 95 °C for 3 min, followed by 40 cycles of denaturation at 95 °C for 5 s and annealing/extension at 65 °C for 30 s. Samples with a threshold cycle (Ct) value ≤35 were considered positive. Synthetic plasmid DNA was used as a positive control, and nuclease-free water served as the negative control. A mare was classified as PCR-positive if at least one collected sample tested positive. Positive samples were not subjected to sequencing or confirmation using an independent molecular assay. Therefore, classification was based on the predefined Ct threshold, assay-specific controls, and the EHV-1-specific real-time PCR result.

In addition to EHV-1 testing, fetal tissues from aborted fetuses and placental samples were examined for other potential infectious causes of abortion by real-time PCR under the same amplification conditions used for EHV-1. Tests for EHV-3, EHV-4, and *Leptospira* spp. yielded negative results. Primer and probe sequences for each agent analysed were listed in Table 2. Bacteriological and fungal cultures of placental and pooled fetal tissue samples were also performed, and no bacterial or fungal agent of diagnostic significance was isolated [21]. Thus, among the infectious agents investigated, EHV-1 was the only agent identified in the abortion cases. The real-time PCR assay was used solely for the detection of EHV-1 DNA. No strain characterization, genotyping, or sequencing was performed; therefore, the detected viruses could not be classified according to neuropathogenicity-associated or other strain-specific markers.

### 2.4. Cytokine ELISAs

Blood samples collected without anticoagulant were centrifuged at 3000 rpm for 10 min to obtain serum. Serum samples were aliquoted and stored at −80 °C until analysis. Serum concentrations of interleukin-2 (IL-2), interleukin-10 (IL-10), interferon-alpha (IFN-α), and interferon-gamma (IFN-γ) were measured using equine-specific ELISA kits (Sunredbio, Shanghai, China) according to the manufacturer’s instructions. For each cytokine, all study samples were analyzed on a single 96-well ELISA plate, thereby minimizing potential inter-plate variability. The cytokine calibration standards supplied with each ELISA kit were analyzed in duplicate to generate the corresponding standard curves, whereas serum samples were analyzed in single wells. Because the available serum from each mare was distributed across four separate cytokine assays, the available serum volume and plate capacity did not permit technical duplicate analysis of all serum samples. Absorbance was measured at 450 nm using a microplate reader (ELx800, BioTek Instruments, Winooski, VT, USA), and cytokine concentrations were calculated from the corresponding standard curves. The manufacturer-specified analytical ranges were 1–300 pg/mL for IL-2, 8–2000 pg/mL for IL-10, 1.5–400 pg/mL for IFN-α, and 3–600 pg/mL for IFN-γ. All cytokine concentrations measured in the study were within the corresponding analytical ranges, and serum samples were analyzed without prior dilution.

The mean coefficients of variation calculated from the duplicate standard measurements were 8.72% for IL-2, 4.92% for IL-10, 5.34% for IFN-α, and 9.97% for IFN-γ. Because serum samples were not analyzed in technical duplicate, sample-specific within-run repeatability could not be directly assessed. Cytokine ratios (IL-10/IL-2 and IFN-γ/IL-10) were calculated as exploratory indicators of the balance between immune regulation and antiviral activity, a concept previously used in viral immunology [22].

**Table 2:** Primer and probe sequences of the agents used in Real-Time PCR.

Agent	Target Region	Primer/Probe Sequence (5′ → 3′)	Reference
EHV-1	gB	Forward (F): GGG-GTT-CTT-AAT-TGC-ATT-CAG-ACC Reverse (R): GTA-GGT-GCG-GTT-AGA-TCT-CAC-AAG Probe (P): (FAM)-TCT-CCA-ACG-AAC-TCG-CCA-GGC-TGT-ACC-(BHQ1)	WOAH Terrestrial Manual (2024) [19]
EHV-4	ORF 17	Forward (F): TAG-CAA-ACA-CCC-ACT-AAT-AAT-AGC-AAG Reverse (R): GCT-CAA-ATC-TCT-TTA-TTT-TAT-GTC-ATA-TGC Probe (P): (FAM)-CGG-AAC-AGG-AAC-TCA-CTT-CAG-AGC-CAG-C-(BHQ1)	WOAH Terrestrial Manual (2017) [23]
EHV-3	gG	Forward (F): GGG-TAT-CGG-CTT-TCT-CAT-CTT-G Reverse (R): CCG-CAG-GAC-GCA-AAC-G Probe (P): (FAM)-TGT-GTC-TCC-TCA-TCG-GCC-TCA-TTG-TCT-(BHQ1)	Barrandeguy et al. (2008) [24]
*Leptospira* spp.	LipL32	Forward (F): AAG-CAT-TAC-CGC-TTG-TGG-TG Reverse (R): GAA-CTC-CCA-TTT-CAG-CGA-TT Probe (P): (FAM)-AAA-GCC-AGG-ACA-AGC-GCC-G-(BHQ1)	Stoddard et al. (2009) [25]

### 2.5. Statistical Analysis

Data distributions were assessed using the Shapiro–Wilk test together with graphical inspection. Considering the small group sizes and the observed distributional characteristics of the data, group differences were evaluated using the Kruskal–Wallis test, followed by Holm-adjusted Mann–Whitney U tests for pairwise comparisons. Two-sided 95% confidence intervals for group means, including cytokine ratios, were calculated using the t distribution. Cohen’s d was calculated as the difference between group means divided by the pooled standard deviation and was reported solely as a descriptive measure of effect size rather than as a basis for statistical inference. Approximate 95% confidence intervals were calculated using the conventional normal-approximation method based on the estimated standard error of Cohen’s d. A *p* value < 0.05 was considered statistically significant. Statistical analyses were performed using SPSS software (version 26.0).

## 3. Results

### 3.1. Detection of EHV-1 DNA

EHV-1 DNA was detected in multiple biological samples collected from PCR-positive mares (Table 3). In the AM group, viral DNA was identified in fetal tissues, placenta, nasopharyngeal swabs, and EDTA blood samples, whereas in the WCS group, positive results were limited to nasopharyngeal swabs and EDTA blood samples. Under the same assay conditions and within a given sample type, lower Ct values indicate a greater amount of detectable EHV-1 target DNA, whereas higher Ct values indicate a smaller amount. Because no absolute quantification standard was used, Ct values were not interpreted as direct measurements of viral load. Ct values varied across sample types, with generally lower values observed in placental and fetal tissues in the AM group, and higher Ct values noted in nasopharyngeal swabs and blood samples, particularly in the WCS group (Table 3). All abortion-associated samples tested negative for EHV-3, EHV-4, and *Leptospira* spp. Bacteriological examination of placental and pooled fetal tissue samples also yielded negative results. EHV-1 was therefore the only infectious agent detected among those investigated in the abortion cases.

### 3.2. Cytokine Concentrations

**Figure 1:**
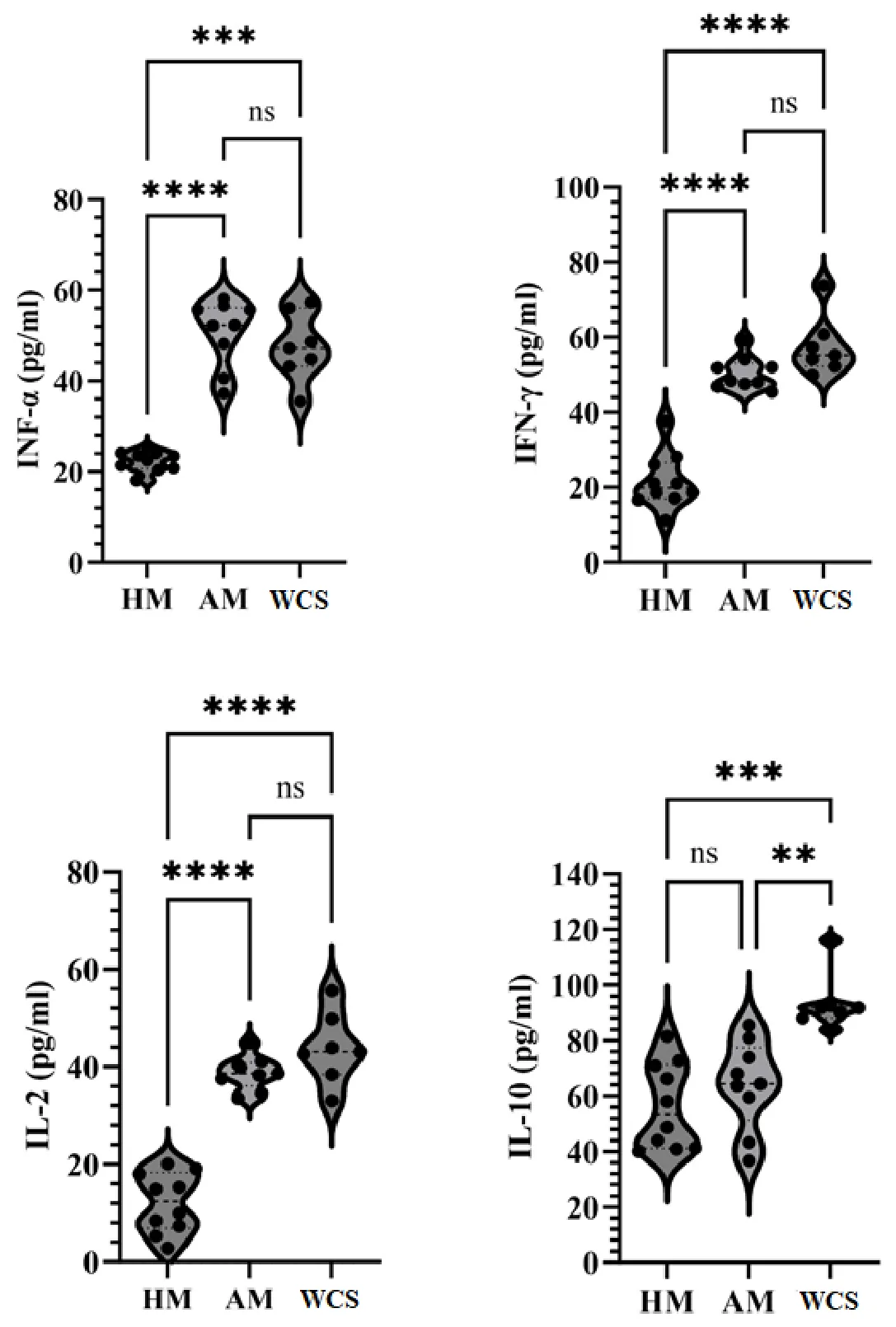
Violin plots showing serum concentrations of IFN-α, IFN-γ, IL-2 and IL-10 in HM, AM and WCS. Individual data points and median values are displayed. Statistical significance between groups is indicated as ns (not significant); ** *p* < 0.01, *** *p* < 0.001 and **** *p* < 0.0001.

Serum cytokine concentrations and derived ratios differed significantly among the study groups (Table 4). Kruskal–Wallis tests showed significant overall group differences for all evaluated variables, including IFN-α, IFN-γ, IL-2, IL-10, IL-10/IL-2, and IFN-γ/IL-10 (all *p* ≤ 0.012). Holm-adjusted pairwise Mann–Whitney U tests showed that IFN-α, IFN-γ, and IL-2 concentrations were significantly higher in the AM group than in the HM group (all *p* < 0.0001). The WCS group also had significantly higher concentrations of IFN-α (*p* < 0.001), IFN-γ (*p* < 0.0001), IL-2 (*p* < 0.0001), and IL-10 (*p* < 0.001) than the HM group. No statistically significant differences in IFN-α, IFN-γ, or IL-2 concentrations were observed between the AM and WCS groups. In contrast, IL-10 concentrations were significantly higher in the WCS group than in the AM group (*p* < 0.01), whereas no statistically significant difference was observed between the HM and AM groups (Figure 1; Table 4).

### 3.3. Cytokine Ratios

**Figure 2:**
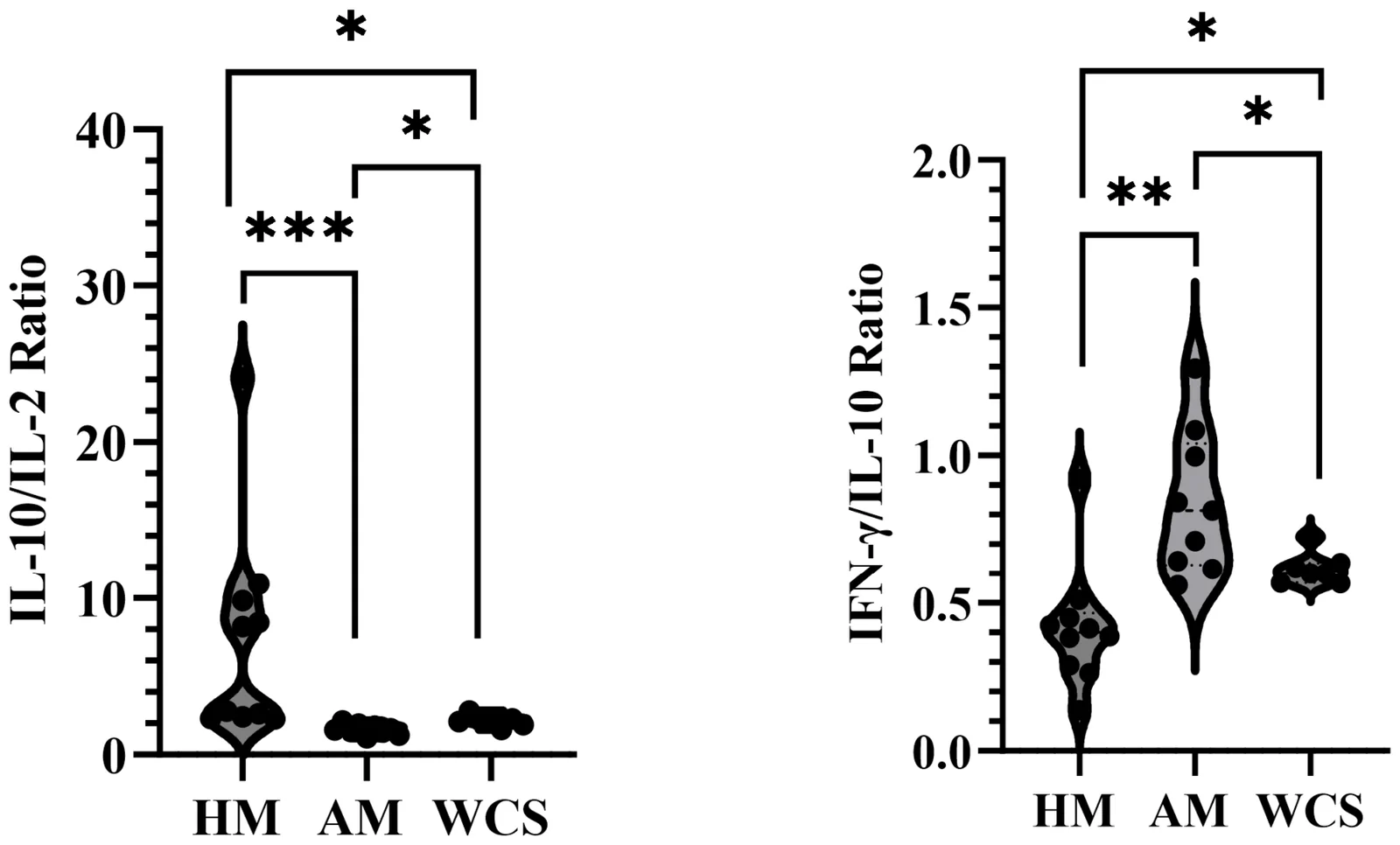
Violin plots of serum cytokine ratios in healthy mares (HM), abortion mares (AM), and PCR-positive mares without clinically relevant signs (WCS). Individual points represent individual mares, and violin shapes provide descriptive summaries of the data distributions. Statistical comparisons were performed using the Kruskal–Wallis test followed by Holm-adjusted pairwise Mann–Whitney U tests. Symbols represent statistica significances: (* *p* < 0.01; ** *p* < 0.001; *** *p* < 0.0001).

Cytokine ratios showed distinct group-specific differences (Table 4). The IL-10/IL-2 ratio differed significantly among the groups (Kruskal–Wallis H = 17.8, *p* < 0.001). Holm-adjusted pairwise comparisons showed that the ratio was significantly higher in HM than in both AM (*p* < 0.001) and WCS (*p* = 0.019), and significantly higher in WCS than in AM (*p* = 0.019; Figure 2). The IFN-γ/IL-10 ratio also differed significantly among the groups (Kruskal–Wallis H = 14.3, *p* < 0.001). Holm-adjusted pairwise comparisons showed that the ratio was higher in AM than in both HM (*p* = 0.005) and WCS (*p* = 0.044), and higher in WCS than in HM (*p* = 0.015; Table 4; Figure 2).

## 4. Discussion

This study demonstrated the presence of detectable EHV-1 DNA in multiple biological matrices from PCR-positive mares. EHV-1 DNA was detected in fetal and placental tissue samples obtained from AM mares, whereas viral DNA was detected in blood and nasopharyngeal swabs from both infected groups. Because fetal and placental samples were unavailable from WCS mares, uteroplacental involvement could not be directly compared between the two infected groups. A recent molecular surveillance study conducted in Türkiye detected EHV-1 DNA in 2 of 152 abortion cases, indicating that the frequency of EHV-1 detection in abortion investigations may vary according to the epidemiological setting, study population, and sampling strategy [26,27]. The Ct values obtained from the different biological samples provide useful, although indirect, information regarding the distribution of detectable EHV-1 DNA. Placental and fetal samples from AM mares yielded lower Ct values than peripheral blood and nasopharyngeal samples, consistent with a greater amount of detectable viral target DNA in tissues directly involved in EHV-1-associated pregnancy loss [28]. Nevertheless, Ct values obtained from different biological matrices should be compared cautiously because matrix composition, nucleic acid extraction efficiency, and sample cellularity may influence amplification results. Furthermore, because the assay was not calibrated against an absolute quantitative standard, Ct values cannot be interpreted as direct measurements of viral load.

Peripheral blood Ct ranges overlapped considerably between AM and WCS mares despite their different clinical outcomes. Because these measurements were obtained from the same biological matrix under the same assay conditions, this overlap suggests that the amount of detectable EHV-1 DNA in peripheral blood at a single sampling point was insufficient to distinguish mares that aborted from PCR-positive mares without clinical signs (WCS). EHV-1 viremia is generally transient, and the amount of viral DNA detected in peripheral blood may vary according to the timing of infection and sampling [28]. Clinical outcome may therefore depend not only on the amount of detectable viral DNA in the circulation but also on viral dissemination to target tissues and the nature of the host immune response. Experimental evidence suggests that pre-existing EHV-1-specific cellular immune responses may influence the control of post-infection viremia and subsequent clinical outcome [22]. Because fetal and placental samples were not available from WCS mares, possible differences in uteroplacental viral involvement between the two infected groups could not be evaluated directly.

The cytokine findings demonstrated substantial innate and cell-mediated immune activation in both EHV-1-positive groups. Serum IFN-α, IFN-γ, and IL-2 concentrations were significantly higher in samples from AM and WCS mares than in HM mares. Type I interferons, including IFN-α, constitute an important component of the early antiviral response by restricting viral replication and promoting antigen presentation [28,29]. Similarly, IFN-γ contributes to macrophage activation and cytotoxic immune responses, whereas IL-2 supports the proliferation and differentiation of activated T lymphocytes [30]. The increased concentrations of these cytokines are therefore consistent with the activation of innate and Th1-associated antiviral responses during EHV-1 infection, as reported previously [31,32].

Importantly, IFN-α, IFN-γ, and IL-2 concentrations did not differ significantly between AM and WCS mares. This finding indicates that substantial antiviral cytokine activation occurred in both infected groups and that the magnitude of these responses alone did not distinguish mares sampled after abortion from PCR-positive mares without clinical signs. In contrast, IL-10 concentrations were significantly higher in WCS mares than in both HM and AM mares. IL-10 limits excessive Th1-associated inflammation and may reduce immune-mediated tissue injury during infection [33,34]. The higher IL-10 concentrations in WCS mares therefore suggest that EHV-1 infection in these mares was associated with a comparatively stronger regulatory component at the time of sampling. This should be interpreted cautiously because the cross-sectional design does not establish that the higher IL-10 concentrations contributed to the maintenance of pregnancy or prevented clinical disease. Alternative explanations for the different outcomes include variation in the timing of infection and sampling, individual host responses, viral dissemination beyond the peripheral circulation, and the extent of uteroplacental involvement. Although higher IL-10 expression has been associated with modulation of disease severity in viral infections [35,36], the present findings demonstrate an association rather than a protective or causal effect.

The cytokine ratios provided additional information regarding the relative balance between inflammatory and regulatory immune activity. The IL-10/IL-2 ratio was highest in HM mares, intermediate in WCS mares, and lowest in AM mares, whereas the IFN-γ/IL-10 ratio showed the opposite pattern. All three groups differed significantly for both ratios. These reciprocal patterns suggest a shift from the relatively regulatory immune environment observed in healthy pregnancy toward a more pro-inflammatory profile in mares sampled after abortion, with WCS mares occupying an intermediate immunological position. Pregnancy requires immune adaptations that maintain fetal tolerance and preserve protection against infection [37], whereas excessive Th1-biased activity has been associated with adverse pregnancy outcomes [38]. Accordingly, the lower IL-10/IL-2 and higher IFN-γ/IL-10 ratios in AM mares are compatible with a stronger inflammatory bias at the time of sampling.

The intermediate ratio values in WCS mares may reflect antiviral immune activation accompanied by a comparatively greater regulatory counterbalance. However, because all AM mares were sampled after fetal expulsion, the observed ratio pattern cannot establish whether the inflammatory imbalance preceded abortion or resulted partly from the acute inflammatory processes associated with fetal expulsion and placental tissue disruption. The cytokine ratios should therefore be regarded as exploratory indicators of immune balance rather than validated markers of pregnancy maintenance. Prospective studies involving serial sampling before clinical outcome are required to determine whether these ratios have prognostic value.

Taken together, the cytokine findings indicate that both infected groups mounted strong innate and cell-mediated antiviral responses, whereas the relative balance between inflammatory and regulatory activity differed among the groups. Mares sampled after abortion had the lowest IL-10/IL-2 ratio and the highest IFN-γ/IL-10 ratio, consistent with a predominantly pro-inflammatory profile at that time. WCS mares had the highest absolute IL-10 concentrations and intermediate values for both ratios, suggesting a comparatively stronger regulatory component despite clear evidence of antiviral immune activation. These observations support an association between clinical presentation and host immune-response balance, but they do not demonstrate that the measured cytokine profiles determined or prevented pregnancy loss [39].

All mares had comparable documented vaccination exposure before sampling. Each mare had received the scheduled inactivated EHV-1/EHV-4 vaccine during the fifth month of gestation; however, sampling occurred before the seventh-month booster, and therefore none of the mares had completed the recommended three-dose gestational vaccination schedule at the time of sampling [40,41]. The fifth-month dose represented the initial dose of this schedule and was intended to initiate baseline protection. Accordingly, the findings should be interpreted cautiously in the context of an incomplete gestational vaccination schedule. Nevertheless, even completion of the recommended three-dose schedule does not provide complete protection against EHV-1 infection or disease, including abortion [17]. Therefore, the occurrence of PCR-positive infection and abortion in this cohort should not be interpreted as definitive vaccine failure. Because all mares had received the same number of vaccine doses before sampling, differences in vaccination exposure are unlikely to account for the observed cytokine differences among the groups. However, the timing of natural infection relative to vaccination, individual variation in vaccine-induced immunity, and the infecting viral genotype were not determined. Furthermore, because unvaccinated mares were excluded according to the predefined eligibility criteria, the study did not include an unvaccinated comparison group. Therefore, the possible influence of vaccination on cytokine responses could not be separated from the immune response to natural infection.

An important limitation of the study was the timing of cytokine measurement in relation to abortion. All AM mares were sampled within 4 h after fetal expulsion, and no pre-abortion serum samples were available for paired analysis. It was therefore not possible to determine whether the observed cytokine profile preceded abortion or reflected, at least in part, the acute inflammatory response associated with fetal expulsion and placental tissue disruption. Consequently, the increased IFN-α, IFN-γ, and IL-2 concentrations and the higher IFN-γ/IL-10 ratio in AM mares should not be interpreted as evidence of a causal role in inducing abortion. Rather, they demonstrate an association between the post-abortion state and a predominantly pro-inflammatory immune profile.

The present findings also do not support the immediate clinical use of cytokine profiling to predict pregnancy outcome. The cross-sectional design, small group sizes, post-abortion sampling of AM mares, and absence of prognostic thresholds, sensitivity, specificity, predictive values, and external validation prevent firm conclusions regarding clinical utility. The observed cytokine patterns and ratios should therefore be considered exploratory immunological findings rather than clinically validated prognostic markers.

Several analytical and molecular limitations should also be acknowledged. Serum samples were analyzed in single wells rather than in technical duplicate; therefore, sample-specific within-run repeatability could not be assessed directly. The coefficients of variation calculated from duplicate standards describe agreement between standard wells and should not be interpreted as estimates of repeatability for individual serum samples. In addition, positive PCR results were not confirmed by sequencing or a second independent molecular assay. Although assay-specific positive and negative controls were included in each run, independent confirmation of the detected EHV-1 DNA was not available.

Viral strain characterization and genotyping of neuropathogenicity-associated or other strain-specific markers were not performed. It was therefore not possible to determine whether AM and WCS mares were infected with the same EHV-1 genotype or whether viral genetic differences contributed to their distinct clinical outcomes and cytokine profiles. Moreover, the small number of PCR-positive mares, the restricted range of peripheral blood Ct values, and the absence of absolute viral quantification precluded a sufficiently powered evaluation of the relationship between detectable viral DNA in blood and serum cytokine concentrations [42]. Another limitation is the analytical cohort’s sample size, which comprised a relatively small number of mares in each. Furthermore, single-point cross-sectional sampling poses inherently diagnostic challenges for viral detection. As demonstrated by Cooper et al. (2026) [43] in their study on EHV-1 shedding in healthy broodmares, longitudinal tracking involving repeated sampling over six time points revealed that 85% of mares tested positive at least once, yet many of these animals yielded negative results during individual sampling events. Consequently, relying on a single sampling time point cannot definitively rule out active shedding or low-level circulating viral DNA. True diagnostic negativity in the healthy control group (HM) cannot be confirmed, raising the possibility that some mares classified as HM may have harbored transient or intermittent viral infection and potentially belonged to the WCS group. Therefore, statistical and immunological comparisons between the HM and WCS groups should be evaluated with caution [43].

Future prospective studies should include serial sampling before clinical outcome, larger independent cohorts, standardized analytical procedures, and external validation of any proposed cytokine thresholds. Combining cytokine profiling with absolute viral quantification and molecular characterization of EHV-1-positive samples would help clarify how viral distribution, viral genotype, and host immune regulation interact to influence reproductive outcome.

## 5. Conclusions

EHV-1 infection in pregnant mares is associated with distinct immune response patterns despite vaccination and standard herd management. Mares sampled after abortion showed a predominantly pro-inflammatory cytokine profile, whereas PCR-positive WCS mares showed a comparatively stronger regulatory immune component at the time of sampling. Serum cytokine profiling, particularly assessment of the IL-10/IL-2 and IFN-γ/IL-10 ratios, may provide a complementary approach for characterizing the balance between regulatory and pro-inflammatory immune responses. Both cytokine ratios differed significantly among the three groups, whereas several individual cytokine concentrations did not differ significantly between the AM and WCS groups. Nevertheless, these ratios should currently be regarded as exploratory immunological indicators rather than validated biomarkers.

## Figures and Tables

**Table 1:** Definition and clinical characteristics of the study groups.

Group	n	EHV-1 PCR Status	Reproductive/Clinical Status	Sampling Time
HM	10	Negative	Healthy pregnant mares	During the outbreak investigation period
AM	9	Positive	Mares that aborted at approximately the sixth month of gestation	Within 4 h after fetal expulsion
WCS	7	Positive	Pregnant mares without clinically relevant signs	During the outbreak investigation period

Abbreviations: HM, healthy mares; AM, abortion mares; WCS, PCR-positive pregnant mares without clinically relevant signs; EHV-1, equine herpesvirus 1.

**Table 3:** EHV-1 PCR positivity and Ct values according to sample type in AM and WCS mares. Data are presented as the number of PCR-positive samples/number tested; median Ct value (minimum–maximum). N.A., not applicable because fetal and placental samples were not available from WCS mares. Ct values are reported for PCR-positive samples only.

Sample Type	AM (n = 9)	WCS (n = 7)
Fetal tissues	9/9; 27.3 (24.3–30.6)	N.A.
Placenta	9/9; 21.3 (19.2–22.7)	N.A.
Nasopharyngeal swab	9/9; 31.1 (28.5–33.2)	7/7; 31.3 (29.6–34.2)
EDTA blood	9/9; 33.6 (30.2–34.8)	7/7; 34.3 (33.7–34.7)

**Table 4:** Serum cytokine concentrations and cytokine ratios in the study groups. Values for the HM, AM, and WCS groups are presented as mean ± standard deviation, with the 95% confidence interval of the group mean shown in square brackets. Pairwise effect sizes are presented as Cohen’s d, with approximate 95% confidence intervals shown in square brackets.

Parameter	HM (n = 10)	AM (n = 9)	WCS (n = 7)	HM vs. AM, Cohen’s d [95% CI]	HM vs. WCS, Cohen’s d [95% CI]	AM vs. WCS, Cohen’s d [95% CI]
**Cytokine Concentrations (pg/mL)**						
IL-2	12.12 ± 6.14 [7.72–16.51] ^a^	38.77 ± 3.41 [36.15–41.40] ^b^	43.79 ± 7.34 [37.00–50.58] ^b^	−5.28 [−7.28–−3.29]	−4.77 [−6.73–−2.81]	−0.92 [−1.95–0.14]
IL-10	56.44 ± 15.47 [45.38–67.51] ^a^	63.95 ± 16.03 [51.63–76.27] ^a^	93.49 ± 10.42 [83.85–103.10] ^b^	−0.48 [−1.39–0.44]	−2.71 [−4.08–−1.34]	−2.12 [−3.39–−0.86]
IFN-α	22.37 ± 2.18 [20.81–23.94] ^a^	50.75 ± 7.36 [45.09–56.41] ^b^	47.55 ± 7.53 [40.58–54.51] ^b^	−5.36 [−7.38–−3.35]	−4.98 [−7.01–−2.95]	0.43 [−0.57–1.43]
IFN-γ	21.62 ± 7.38 [16.33–26.90] ^a^	50.41 ± 4.36 [47.06–53.76] ^b^	57.65 ± 7.94 [50.31–65.00] ^b^	−4.68 [−6.50–−2.87]	−4.73 [−6.68–−2.78]	−1.18 [−2.26–−0.10]
**Cytokine Ratios**						
IL-10/IL-2	7.39 ± 6.84 [2.50–12.28] ^a^	1.63 ± 0.33 [1.38–1.89] ^c^	2.18 ± 0.36 [1.84–2.51] ^b^	1.16 [0.17–2.14]	0.98 [−0.04–2.01]	−1.56 [−2.71–−0.42]
IFN-γ/IL-10	0.42 ± 0.21 [0.27–0.57] ^c^	0.84 ± 0.24 [0.65–1.03] ^a^	0.62 ± 0.05 [0.57–0.67] ^b^	−1.88 [−2.98–−0.78]	−1.22 [−2.28–−0.16]	1.19 [0.11–2.27]

Different superscript letters within the same row indicate statistically significant differences between groups based on Holm-adjusted pairwise Mann–Whitney U tests. Cohen’s d was calculated as the mean of the first group minus the mean of the second group; therefore, positive values indicate higher concentration in the first group; negative values indicate higher concentrations in second group. Abbreviations: HM, healthy mares; AM, abortion mares; WCS, PCR-positive mares without clinically relevant signs; CI, confidence interval; SD, standard deviation.

## Data Availability

The raw data supporting the conclusions of this article will be made available by the authors on request.
